# Altered Gluteus Maximus and Latissimus Dorsi Activation in Subacromial Impingement Syndrome: Implications for Kinetic Chain Dysfunction

**DOI:** 10.3390/jcm15166325

**Published:** 2026-08-16

**Authors:** Nada M. Emara, Sohair S. Rezkallah, Osama R. Abdelraouf, Mohamed M. Ahmed, Zizi M. Ibrahim, Nouf H. Alkhamees, Aya Abdelhamid Khalil

**Affiliations:** 1 Department of Biomechanics, Faculty of Physical Therapy, Pharos University, Alexandria 21523, Egypt; nada.emara@pua.edu.eg; 2 Department of Basic Science, Faculty of Physical Therapy, Cairo University, Giza 12613, Egypt; sohair.shehata@pt.cu.edu.eg; 3 Physical Therapy Program, Batterjee Medical College, Jeddah 21442, Saudi Arabia; pt4.jed@bmc.edu.sa; 4 Department of Physical Therapy, College of Nursing and Health Sciences, Jazan University, Jazan 45142, Saudi Arabia; 5 Department of Basic Sciences in Physical Therapy, Faculty of Physical Therapy, Beni-Suef University, Beni Suef 62521, Egypt; 6 Department of Rehabilitation Sciences, College of Health and Rehabilitation Sciences, Princess Nourah bint Abdulrahman University, P.O. Box 84428, Riyadh 11671, Saudi Arabia; zmibrahim@pnu.edu.sa (Z.M.I.); nhalkhamees@pnu.edu.sa (N.H.A.); 7 Department of Biomechanics, Faculty of Physical Therapy, Cairo University, Giza 12613, Egypt; aya.abdelhamied@pt.cu.edu.eg

**Keywords:** subacromial impingement syndrome, posterior oblique sling, kinetic chain dysfunction, surface electromyography, gluteus maximus, latissimus dorsi

## Abstract

**Background/Objectives**: Subacromial impingement syndrome (SIS) may be associated with altered kinetic-chain mechanics above and below the shoulder. The posterior oblique sling integrates the gluteus maximus and latissimus dorsi through established fascial and neuromuscular connections, yet empirical evidence examining its relevance in SIS remains limited. Therefore, this cross-sectional observational study aimed to investigate the surface electromyographic (sEMG) activity of the latissimus dorsi (LD) and contralateral gluteus maximus (GM) and evaluate their associations with pain, disability, and shoulder range of motion (ROM) in young adults with SIS. **Methods:** Sixty sedentary young adults with SIS and matched controls were assessed using the Numeric Pain Rating Scale, Arabic QuickDASH, twin-axis electrogoniometer, and sEMG of the gluteus maximus and latissimus dorsi. **Results:** The SIS group had significantly lower GM activity (23.96 ± 3.54% MVIC for SIS and 34.49 ± 4.16% MVIC for controls) and significantly higher LD activity (118.63 ± 24.87% MVIC for SIS and 75.52 ± 25.93% MVIC for controls) (*p* = 0.001). In addition to these differences, the SIS group exhibited significantly less ROM in all directions (flexion, extension, abduction, and rotation) and greater disability and pain intensity (both *p* = 0.001) than controls. Correlations between sEMG activity and clinical outcome measures in the SIS group were weak and not statistically significant. Discriminant logistic regression indicated that both GM and LD sEMG were significant discriminative variables of group membership, with GM underactivity showing the greater discriminative value. **Conclusions:** These results indicate an altered neuromuscular activation pattern observed in participants with SIS, characterized by decreased GM and increased LD sEMG activity. They may justify further investigation of kinetic-chain-informed rehabilitation, with attention to both hip/lumbopelvic function and shoulder-based treatment.

## 1. Introduction

Shoulder pain is the third most common musculoskeletal complaint in primary care, and its clinical presentation varies widely among individuals. Half of these patients still experience shoulder pain six months after the initial visit to the primary care physician [1]. Previous research estimates that shoulder pain affects 7% to 34% of individuals, with many cases linked to subacromial impingement syndrome (SIS), which is influenced by multiple mechanical, biological, neuromuscular, and load-related factors. Repetitive overhead or shoulder-level activities may contribute to symptom development, especially among young adults, but they also should not be presented as the sole cause [2,3,4]. It was reported that SIS constitutes almost 44–65% of shoulder complaints in clinical settings [5], although patients presenting with signs and symptoms of subacromial pain syndrome often have mixed and concomitant diagnoses, highlighting the diagnostic heterogeneity of this condition [6,7].

When left untreated, SIS can lead to progressive degenerative changes in the shoulder joint, ongoing pain, and other conditions, including rotator cuff tears [8].

Recent evidence suggests that myofascial factors contribute meaningfully to subacromial impingement syndrome [9]. Myofascial trigger points and associated dysfunction in the shoulder and scapular muscles can also affect the trunk and lower extremities through altered postural control and disruptions of the kinetic chain. Impaired scapular stability and muscle imbalances can lead to compensatory changes in thoracic spine posture, promoting increased kyphosis and decreased trunk extension, which negatively affect overall posture and balance and contribute to inefficient movement patterns [10,11].

Fascia connects muscles throughout the body and supports movement and clinical function [12]. Four main myofascial slings, anterior oblique, posterior oblique, deep longitudinal, and lateral, help transfer forces, improve stability, and enhance coordination and strength [13,14,15]. Training these sling systems can improve coordination, stability, performance, and injury prevention [16].

Particularly, the posterior oblique sling, primarily involving the latissimus dorsi, contralateral gluteus maximus, and thoracolumbar fascia, is specifically essential in force closure across the sacroiliac joint and load transfer through the lumbopelvic area to the shoulders during gait and rotational activities [15,17]. Exercising the gluteus maximus has been suggested to be beneficial in modifying the pattern of sling activation and improving dynamic lumbopelvic stability [18,19].

By supporting scapular stability, the lumbopelvic hip muscles effectively contribute to shoulder movement [20]. On the other hand, the latissimus dorsi, in addition to being a primary shoulder mover that aids in the adduction, extension, and internal rotation of the humerus, also stabilizes the scapulothoracic and glenohumeral joints [21]. Dysfunction of the latissimus dorsi can significantly alter shoulder kinematics and lead to compensatory muscle demands [22].

To date, most electromyographic studies in SIS have focused on local shoulder muscles, particularly the rotator cuff and scapular stabilizers, because these structures are directly implicated in shoulder pain and impaired movement. However, the role of more proximal kinetic-chain structures, such as the posterior oblique sling, has received far less attention, partly reflecting the lack of a more holistic approach to assessment and treatment.

Despite the known anatomical and functional links within the posterior oblique sling, the role of gluteus maximus and latissimus dorsi activation in SIS remains unclear. In particular, it is unknown whether EMG activity in these muscles is associated with pain, disability, or shoulder ROM in patients with SIS.

This study, therefore, aims to assess electromyographic (EMG) activity of the latissimus dorsi and gluteus maximus in patients with shoulder impingement syndrome. Additionally, it seeks to correlate these EMG activities with pain intensity, dysfunction, and shoulder range of motion. It also aims to determine whether EMG measurements of the LD and GM separately explain variance in clinical outcomes in patients with SIS. It was hypothesized that individuals with SIS would demonstrate altered activation of the gluteus maximus and latissimus dorsi compared with healthy controls, with exploratory analysis of potential associations with pain, disability, and shoulder range of motion.

## 2. Materials and Methods

### 2.1. Study Design

This was an observational cross-sectional study conducted from October 2024 to March 2025. The Ethics Committee of the Faculty of Physical Therapy at Cairo University (P.T.REC/012/005452) approved the study, ensuring all procedures adhered to the latest version of the Declaration of Helsinki.

### 2.2. Participants

Thirty sedentary adults (18 females, 12 males) aged 18 to 25 years with unilateral subacromial shoulder pain [23] referred from outpatient physiotherapy clinics, and 30 healthy controls participated in this study. Healthy controls were recruited from the same community as the SIS participants through convenience sampling and were matched to the SIS group for age, sex, BMI, and hand dominance. This age range was chosen to ensure a homogeneous sample of young adults and to minimize age-related confounding factors that could affect shoulder function and EMG measurements.

To be eligible [24,25], participants had to report unilateral shoulder pain for at least 3 months during arm elevation or overhead activity and demonstrate at least two positive findings from a predefined clinical examination cluster. This cluster included the Neer sign, Hawkins–Kennedy sign, a painful arc of active shoulder abduction between 60° and 120°, and pain or weakness during the empty-can or resisted external rotation tests. These tests were used as part of a clinical screening approach to identify a subacromial pain/impingement presentation rather than to confirm a specific structural lesion. Participants were also required to report at least 3/10 pain on the VAS during arm elevation and to achieve at least 90 degrees of shoulder abduction to ensure reliable EMG and ROM testing.

The study exclusion criteria [25,26] included participation in formal fitness or resistance training programs, major shoulder trauma resulting in full- or partial-thickness rotator cuff tears, biceps tendinopathy, acromioclavicular joint pathology, or clinically significant labral lesions, shoulder surgery, previous cervical or lumbar spine surgery, complete rotator cuff tears, adhesive capsulitis, glenohumeral instability, cervical radiculopathy with radiating arm pain and sensory or motor deficits, systemic inflammatory or rheumatologic diseases, known neuromuscular disorders, unmanaged metabolic conditions, and severe low back or pelvic girdle pain that could interfere with EMG assessment of the posterior oblique sling. For technical reasons related to EMG signal quality and reliable electrode placement, participants with a body mass index above 30 kg/m^2^ were excluded [27]. Physically active individuals were excluded to minimize the potential influence of training-related neuromuscular adaptations on EMG activation patterns and to improve sample homogeneity [28].

**Figure 1:**
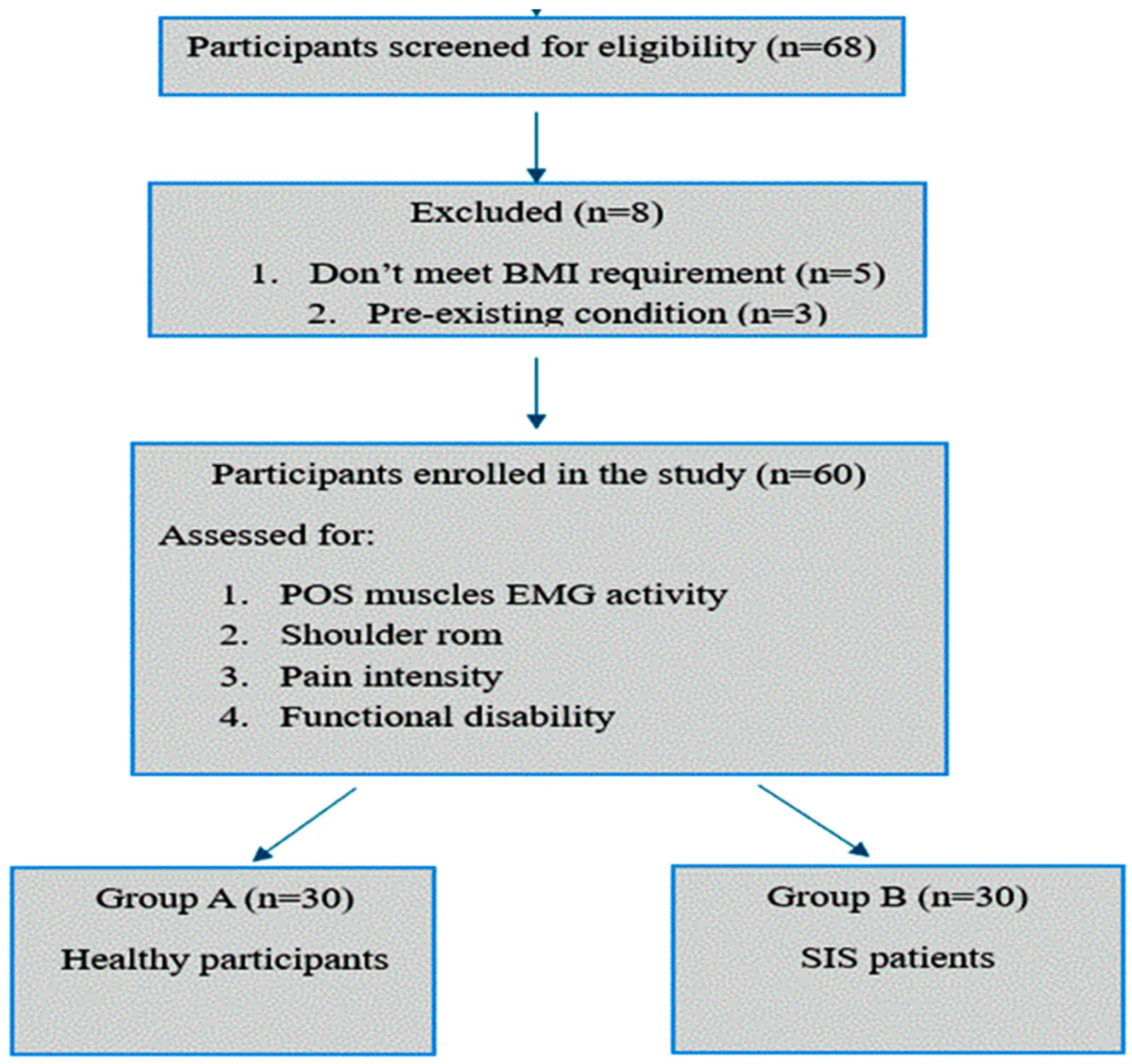
Flowchart of the study.

After being fully informed about the study procedures, potential risks, and anticipated benefits, all participants provided written consent. The progression of participants throughout the study is illustrated in Figure 1.

### 2.3. Sample Size

Sample size was calculated a priori using G*Power (version 3.1.9.3; Universität Düsseldorf, Düsseldorf, Germany) based on the primary hypothesis that participants with subacromial impingement syndrome (SIS) would demonstrate altered normalized gluteus maximus (GM) sEMG activity, expressed as a percentage of maximum voluntary isometric contraction (MVIC), compared to healthy controls. Because the primary objective was to compare the mean GM sEMG activity between two independent groups, an independent-samples t-test was selected for the power analysis. Assuming a two-tailed significance level of α=0.05, statistical power of 1−β=0.60, and a moderate standardized effect size of d=0.50, derived from prior EMG literature in SIS and related shoulder pain studies. This yielded a minimum required sample size of 60 participants, or 30 participants per group [29,30,31]. The correlation and regression analyses were exploratory and were not used to determine the sample size.

### 2.4. Assessment Procedures

Following the Clinical measurement guidelines, it is recommended to progress from the least to the most provocative tests [32]. Accordingly, the assessment order during the study was standardized as follows: pain intensity, perceived dysfunction/disability, Shoulder ROM, and finally EMG of the gluteus maximus and latissimus dorsi. This order minimizes carry-over or fatigue effects during assessment.

Initially, all participants were assessed for demographic data, including age, height, weight, body mass index [BMI], and arm dominance. Arm dominance was determined using the Edinburgh Handedness Inventory (EHI), which, with its validated 10-item short form, is the standard self-report tool for determining dominant arm preference in research. It evaluates tasks such as writing, throwing, and using utensils, in which participants indicate the strength of their right/left preference [33]. Blinding was not feasible because the assessor necessarily observed the participant’s shoulder status and testing procedures during ROM and sEMG data collection, which could reveal group allocation in a cross-sectional study. To minimize bias, we therefore standardized the assessment protocol across all participants.

#### 2.4.1. Pain Intensity

The Arabic version of the Numeric Pain Rating Scale was used to measure pain intensity (NPS), which consists of 10 points. The participants were asked to place a circle around the number on the scale, from 0 (no pain) to 10 (pain as bad as it could be), that most accurately reflected their current perceived level of shoulder pain during overhead arm elevation. A single rating was obtained and used in the analysis. The Arabic NPRS has proven reliable and valid for assessing musculoskeletal pain [34].

#### 2.4.2. Shoulder Function and Disability

The Arabic version of the QuickDASH questionnaire (QuickDASH-Arabic) was used to determine shoulder function and upper-limb disability [35]. The respondents completed the 11-item instrument as instructed by the developers. The QuickDash score was computed by dividing the sum of the scored items by the number of rated items to obtain the average item score, then subtracting one from it and multiplying by 25 to get a total score of 0 to 100, with a higher score indicating greater disability [36]. Excellent internal consistency, test–retest reliability, and good construct validity of the Arabic QuickDASH have been observed in patients with upper-extremity musculoskeletal problems [37].

#### 2.4.3. Shoulder Range of Motion (ROM)

A twin-axis electrogoniometer (SG150, manufactured by Biometrics Ltd., Gwent, UK) was used to measure shoulder range of motion. The trunk was supported to minimize compensatory movements. Participants actively moved the shoulder into flexion, extension, abduction, adduction, external rotation, and internal rotation to their end range, following standardized verbal instructions and a fixed testing sequence. The examiner monitored the trunk movement and made corrections as needed. Three trials were performed in standardized positions, and their average was calculated. The shoulder ROM measurements have been described in detail in the previous literature [38,39].

#### 2.4.4. Surface Electromyography (sEMG)

Surface electromyography (sEMG; Neuro-EMG-Micro-2, Neurosoft, Russia) was used to record muscle activity from the latissimus dorsi (LD) on the symptomatic shoulder side and the contralateral gluteus maximus (GM). EMG signals were sampled at 1000 Hz using a 16-bit analog-to-digital converter. To reduce motion artifact and power-line noise, a band-pass filter of 20–450 Hz and a 50 Hz notch filter were applied during acquisition.

**Figure 2:**
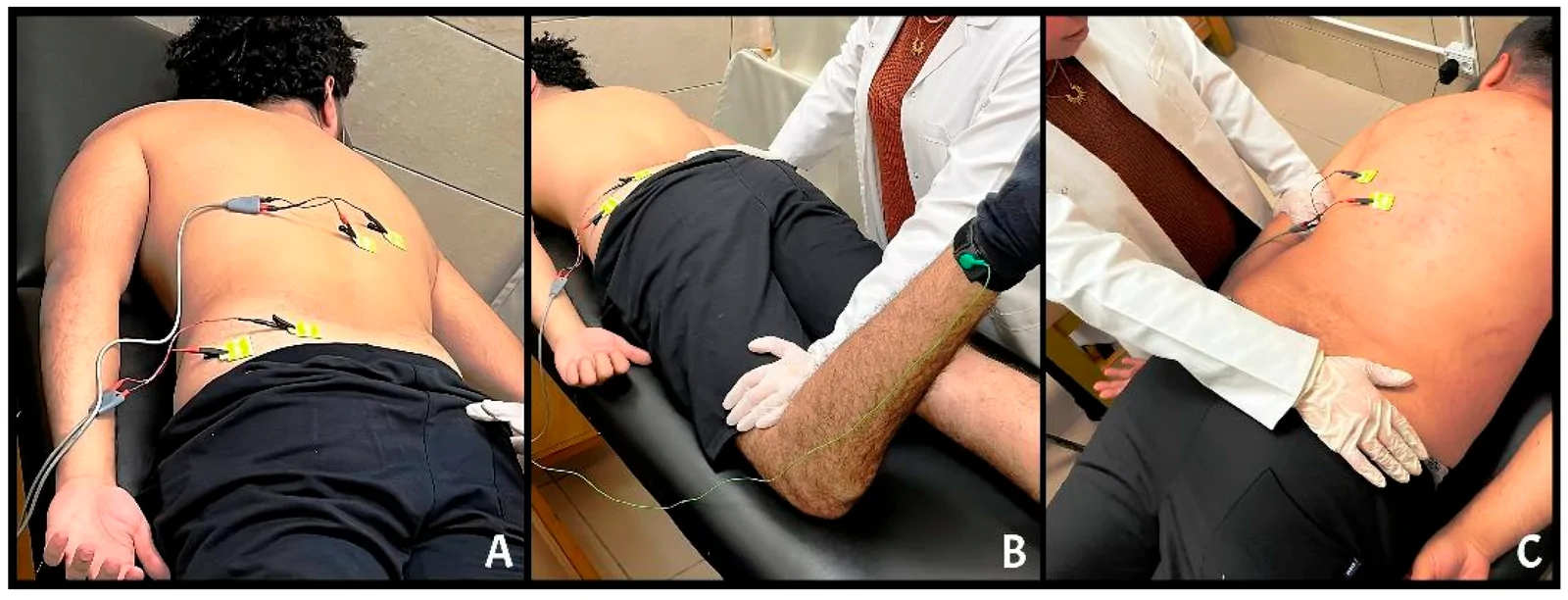
(**A**) Electrode placement, (**B**) gluteus maximus MVIC, (**C**) Latissimus dorsi MVIC.

Bipolar electrodes were positioned over the belly of each muscle according to SENIAM recommendations [40]. As shown in Figure 2A, for the GM, the electrodes were placed at 50% of the line between the sacral vertebrae and the greater trochanter, with the pair oriented parallel to the line joining the posterior superior iliac spine and the midpoint of the posterior thigh [41]. For the LD, electrodes were placed approximately 4 cm inferior to the inferior angle of the scapula and 2 cm medial to the posterior axillary line, oriented parallel to the muscle fibers [42]. The interelectrode distance was maintained at 20 mm, and the skin was prepared before electrode placement.

sEMG was recorded during a standardized experimental task. Participants performed active shoulder elevation in the scapular plane while simultaneously extending the contralateral hip to mimic combined posterior oblique sling activation. The elbow remained extended, and the thumb was directed upward. The arm was elevated from 60° to approximately 120° and then returned to the starting position at a standardized movement speed [19,29,31]. The LD was assessed on the symptomatic shoulder side, which was the dominant side in 28 participants and the nondominant side in 2 participants. The contralateral GM was assessed according to the posterior-oblique-sling pairing. The healthy control group was assessed using the same shoulder–hip pairing rule to minimize dominance-related bias. Three 5 s trials were recorded for each task, with a 5 s rest interval between trials [43]. During data collection, participants received standardized verbal instructions and completed practice trials before recording.

The recorded sEMG traces were visually inspected for prominent artifacts before RMS calculation. Trials showing obvious artifact contamination within the analyzed recording period were excluded in their entirety; isolated portions of contaminated trials were not selectively removed. RMS was calculated over the complete 5 s recording of each retained trial, and the mean RMS across the valid trials (at least two trials) was used for subsequent normalization and statistical analysis. The EMG signal was then processed by calculating the root mean square (RMS) using a 100 ms moving window to obtain the linear envelope [44,45]. The RMS amplitude from each of the three recorded trials was averaged, and the resulting mean RMS value for the experimental movement was normalized to the corresponding muscle-specific maximum voluntary isometric contraction (MVIC) and expressed as %MVIC.

Separate MVIC trials were used only for normalization. GM MVIC was obtained in prone lying with hip extension against manual resistance and the knee flexed to 90° (Figure 2B), whereas LD MVIC was obtained in prone lying with the arm alongside the body and the shoulder internally rotated, while resisting an attempt to raise the shoulder toward the ceiling (Figure 2C). Three 5 s MVIC trials were recorded with 5 s rest intervals, and the average RMS value across the trials was used as the normalization reference [46].

**Figure 3:**
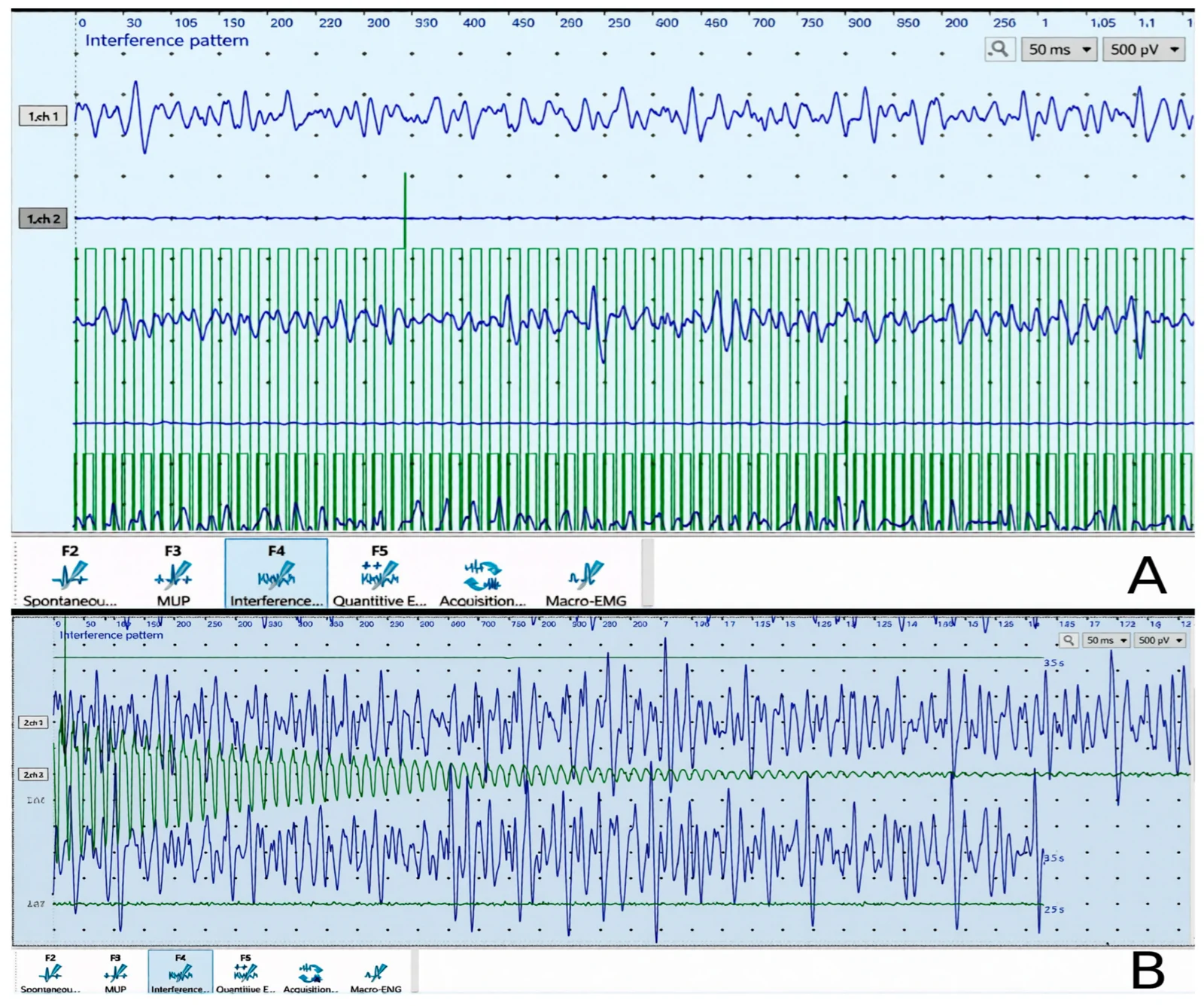
Representative sEMG recording during the analyzed posterior-oblique-sling contraction (**A**) raw, (**B**) normalized.

A representative recording of the experimental contraction is shown in Figure 3. The figure displays simultaneously recorded LD and contralateral GM signals, including the raw sEMG traces (Figure 3A) and the corresponding RMS-derived normalized envelopes expressed as %MVIC (Figure 3B).

### 2.5. Statistical Analysis

Statistical analyses were performed using IBM SPSS Statistics version 25 (IBM Corp., Armonk, NY, USA), with statistical significance set at a two-tailed *p* < 0.05. Continuous variables are presented as mean ± SD. Data normality was assessed using the Shapiro–Wilk test and visual inspection of histograms and Q–Q plots, and homogeneity of variance was evaluated using Levene’s test. Between-group differences in demographic characteristics, normalized EMG activity, ROM, pain, and disability were analyzed using independent-samples *t*-tests, and standardized mean differences were reported as Hedges’ g with 95% confidence intervals. Gender distribution and hand dominance were compared using the chi-square test.

Within the SIS group, Pearson’s correlation coefficient was used to examine associations between EMG activity and pain, disability, and ROM. Discriminant logistic regression was then used to determine whether LD and GM EMG activity explained variance in group membership, and model fit, discrimination, and calibration were assessed using the relevant statistics. Effect-size information was reported as standardized mean differences for between-group comparisons, Pearson’s r for correlations, and odds ratios with 95% confidence intervals for regression models. Effect-size interpretation followed physiotherapy-specific guidelines [47].

## 3. Results

### 3.1. Participant Characteristics

The analysis included 60 participants, 30 healthy individuals, and 30 patients with shoulder impingement syndrome (SIS). Both groups were comparable in age (*p* = 0.33), height (*p* = 0.29), and body mass index (*p* = 0.23), with no significant differences between their means. Moreover, there were no statistically significant between-group differences in gender distribution and hand dominance (χ^2^ = 0 and *p* = 1).

### 3.2. Between-Group Comparisons (Table 1)

#### 3.2.1. EMG Activity

Of the 720 planned muscle-trial recordings, 14 (1.9%) were excluded because of prominent artifacts. These exclusions were distributed across the SIS and control groups and across LD and GM recordings, without a systematic pattern of missingness. RMS values were calculated from the complete retained trials, and the mean of the valid trials was used for subsequent normalization and statistical analysis.

**Table 1:** Between-group comparisons.

Outcome Measures	Variable	Healthy Mean ± SD	SIS Mean ± SD	Mean Difference	95% CI	*p*-Value	Hedges’ g
EMG Activity	Gluteus Maximus EMG (% MVIC)	34.49 ± 4.16	23.96 ± 3.54	5.32	4.16–6.48	0.001 *	2.31
	Latissimus Dorsi EMG (% MVIC)	75.52 ± 25.93	118.63 ± 24.87	43.11	29.9–56.2	0.001 *	1.59
Shoulder ROM	Flexion (degrees)	169.33 ± 5.25	118.8 ± 19.89	50.53	43.0–58.1	0.001 *	3.12
	Extension (degrees)	45.47 ± 3.22	32.43 ± 4.75	13.04	11.0–15.2	0.001 *	3.06
	Abduction (degrees)	168.1 ± 5.5	113.83 ± 13.79	54.27	48.8–59.7	0.001 *	4.32
	External Rotation (degrees)	87.23 ± 2.16	58.83 ± 5.53	28.4	26.2–30.57	0.001 *	6.51
	Internal Rotation (degrees)	66.7 ± 3.09	40.37 ± 5.8	26.33	23.9–28.74	0.001 *	5.42
Pain intensity	Pain (Numeric Pain Scale)	0	5.7 ± 0.9			0.001 *	
Function	QuickDASH Score	0	55.86 ± 6.42			0.001 *	

EMG: Electromyography; MVIC: Maximum Voluntary Isometric Contraction; ROM: range of motion; MD: Mean Difference; CI: Confidence Interval. All values are mean ± SD (standard deviation). * *p*-value < 0.05 indicates a significant difference.

The gluteus maximus EMG activity (% MVIC) was significantly lower in the SIS group than in the healthy controls. The mean activation was 34.49 ± 4.16% MVIC in healthy participants, compared with 23.96 ± 3.54% MVIC in patients with SIS. The mean difference between groups was 5.32 (95% CI: 4.16–6.48) with a highly significant group effect (*p* = 0.001) and a large effect size (Hedges’ g = 2.31). On the contrary, latissimus dorsi EMG was measured much higher in the SIS group. The mean activation of healthy participants was 75.52 ± 25.93% MVIC, whereas in SIS patients, it was 118.63 ± 24.87% MVIC. The average difference was 43.11 (95% CI: 29.9–56.2), with a *p*-value of 0.001 and a Hedges’ g of 1.59.

#### 3.2.2. Shoulder Joint Range of Motion

The mean range values ± SD for the healthy participant group were 169.33 ± 5.25, 45.47 ± 3.22, 168.1 ± 5.5, 87.23 ± 2.16, and 66.7 ± 3.09 degrees for shoulder flexion, extension, abduction, external rotation, and internal rotation, respectively. The SIS group had ranges of 118.8 ± 19.89, 32.43 ± 4.75, 113.83 ± 13.79, 58.83 ± 5.53, and 40.37 ± 5.8 degrees for the same movements. The mean differences were 50.53 (95% CI: 43 to 58.1) for joint flexion, 13.04 (95% CI: 11 to 15.2) for extension, 54.27 (95% CI: 48.8 to 59.7) for abduction, 28.4 (95% CI: 26.2 to 30.57) for external rotation, and 26.33 (95% CI: 23.9 to 28.74) for internal rotation. The statistical significance was *p* = 0.001 in all shoulder joint movement directions.

#### 3.2.3. Pain and Upper-Limb Function

While the healthy group experienced no pain or disability, the SIS group reported pain of 5.7 ± 0.9 on the numeric pain scale and dysfunction of 55.86 ± 6.42, as measured by the QuickDash, with *p* = 0.001 for both outcomes.

### 3.3. Correlation Analyses (Table 2)

EMG activity of the gluteus maximus in the SIS group was positively correlated with pain intensity (r = 0.33, *p* = 0.07) and shoulder abduction range (r = 0.355, *p* = 0.054) on a small-to-moderate level, but neither correlation was significant. Other shoulder movement correlations and functional scores were weak and non-significant. EMG activity of the gluteus maximus in the healthy control group did not show any significant correlations with pain, functional outcomes, or ROM variables (all *p* > 0.05). Latissimus dorsi EMG activity also showed a weak, non-significant correlation with pain, functioning, and ROM in the SIS group (all *p* > 0.05). In healthy subjects, however, activation of the latissimus dorsi showed a significant positive relationship with shoulder abduction range (r = 0.534, *p* = 0.002). The remaining correlations were statistically non-significant.

**Table 2:** Correlation analyses.

Muscle	Variable	SIS Group r	SIS Group *p*	Control Group r	Control Group *p*
Gluteus max EMG activity	Pain	0.33	0.07	-	-
	QDASH	0.09	0.63	-	-
	Flexion	−0.092	0.629	0.088	0.644
	Extension	−0.107	0.574	0.047	0.805
	Abduction	0.355	0.054	0.110	0.562
	External rotation	0.193	0.308	−0.085	0.655
	Internal rotation	0.047	0.806	0.120	0.529
Latissimus dorsi EMG activity	Pain	0.178	0.347	-	-
	QDASH	−0.126	0.506	-	-
	Flexion	−0.110	0.561	0.218	0.247
	Extension	−0.020	0.918	−0.130	0.493
	Abduction	0.099	0.604	0.534	0.002 *
	External rotation	0.250	0.182	−0.150	0.429
	Internal rotation	0.344	0.063	−0.166	0.381

EMG: Electromyography; QDASH: QuickDASH; r: correlation coefficient; * *p*-value < 0.05 indicates a significant difference.

### 3.4. Regression Analyses (Table 3)

Binary logistic regression with gluteus maximus (GM) EMG activity showed that GM was a strong discriminator between SIS patients and healthy controls. The model was significant ($\chi^{2}$(1) = 11.12, *p* = 0.001) and explained 85.5% of the variation in group classification (Nagelkerke R^2^ = 0.855). Model calibration was excellent (Hosmer–Lemeshow, $\chi^{2}$(8) = 2.064, *p* = 0.979). Higher GM activity was associated with lower odds of being classified as a patient (B = −2.007, OR = 0.134, 95% CI 0.041–0.437).

**Table 3:** Regression analysis.

Discriminative Variable	Coeff. (B)	SE	z-Value (Wald)	*p*-Value	OR (95% CI)
EMG Latissimus dorsi	0.051	0.012	16.85	<0.001 *	1.052 (1.027–1.078)
EMG Gluteus max	−2.007	0.602	11.12	0.001 *	0.134 (0.041–0.437)

EMG: Electromyography; SE: standard error; CI: Confidence Interval; * *p*-value < 0.05 indicates a significant difference.

A second logistic regression model using latissimus dorsi (LD) EMG activity was also significant ($\chi^{2}$(1) = 16.85, *p* < 0.001) and explained 49.0% of the variation in patient classification (Nagelkerke R^2^ = 0.490). Model fit was acceptable (Hosmer–Lemeshow, $\chi^{2}$(8) = 11.34, *p* = 0.183). Higher LD activity increased the odds of being classified as a patient (B = 0.051, OR = 1.052, 95% CI 1.027–1.078).

ROC analysis showed good discriminatory ability for LD activity (AUC = 0.853) and excellent discriminatory ability for GM (AUC = 0.977). Overall, these findings suggest that reduced GM activity and increased LD activity may help distinguish SIS from healthy controls in this sample, with GM showing the stronger discriminative value once the direction of association is considered.

## 4. Discussion

The current study investigated the electromyographic behavior of two essential components of the posterior oblique sling (POS): the gluteus maximus (GM) and the latissimus dorsi (LD). It also examined the relationship and discriminative value of altered electromyographic behavior of these muscles with pain, upper-limb dysfunction, and reduced shoulder mobility in individuals with subacromial impingement syndrome (SIS).

Significant results were observed. First, the SIS patients showed significantly reduced GM activation, elevated LD activity, impaired shoulder joint movement in all planes, increased pain, and upper-limb disability compared with healthy controls. Second, in the SIS cohort, although EMG activity of the studied muscles was not significantly related to clinical measures, both LD and GM activity showed significant discrimination between groups, with GM underactivation being the more discriminative predictor. These findings indicate differences in neuromuscular behavior that may involve the kinetic chain in persons with SIS.

The primary outcomes of this study were sEMG activity of the gluteus maximus and latissimus dorsi, whereas pain, disability, and shoulder ROM were secondary clinical variables used to explore their associations with EMG activity. Given the number of statistical tests performed, the correlation and regression analyses were considered exploratory, and small effects were interpreted cautiously in accordance with physiotherapy-specific effect-size guidelines [47].

Reduced GM activation and increased LD activity may reflect altered neuromuscular recruitment in SIS; however, the cross-sectional design does not allow determination of whether one pattern caused the other. The weak correlations suggest these EMG changes are not directly tied to symptom severity, but they may still be useful for identifying the SIS pattern. Clinically, the findings possibly support considering both shoulder and lumbopelvic factors in rehabilitation. However, normalized sEMG amplitude reflects muscle activation patterns rather than direct muscle strength or force output; therefore, the present findings should be interpreted as indicating altered neuromuscular recruitment rather than as direct evidence of weakness or dysfunction.

The observed decrease in GM activation in our study aligns with the growing body of literature indicating that impairment of lumbopelvic control can modify shoulder mechanics through interrelated myofascial pathways and compensatory trunk motions. The present results add to the concept reported in previous studies that dysfunctional GM recruitment significantly affects pelvic stability, alters trunk rotation mechanisms, and increases dependence on proximal chain muscles, as reflected in arm elevation kinematics [12,17,48]. These findings are consistent with the possibility of altered load transfer between the pelvis and shoulder; however, scapular kinematics and decreased subacromial space were not directly measured in this study. All these proposed changes are characteristic of SIS pathomechanics [3,10].

The high LD activation in SIS patients provides further insight into compensatory neuromuscular responses. The LD is a strong humeral depressor and internal rotator, and is integral to scapulothoracic stability [21,22]. Increased LD activity has been reported in some shoulder disorders with dysfunctional scapular upward rotation or overuse of secondary stabilizers [49]. This hyperactivity may represent an adaptive response to reduced GM contribution or inadequate functioning of the scapular stabilizers. In addition, increased LD demand may contribute to altered humeral head mechanics commonly observed in SIS and worsen impingement symptoms. Recent imaging and biomechanical research confirm that LD is dynamically involved in the control of scapular movement during arm elevation, and excessive LD activation may lead to maladaptive scapular kinematics, which can cause pain and dysfunction [50,51].

Although significant differences between groups were observed, the correlation between EMG activity and clinical variables in the SIS group was generally weak. This underscores the multifactorial nature of SIS etiology, in which a combination of structural, neuromuscular, biomechanical, and perceptual factors contributes to symptoms rather than a single determinant. Moreover, neuromuscular adaptations may precede symptom severity or persist even as symptoms fluctuate, making it challenging to observe linear relationships in cross-sectional research designs. Nevertheless, the finding that EMG activity, particularly GM underactivation, has discriminative ability to separate groups within this study indicates that it could be considered a potentially useful discriminative signal in this sample. The ROC analysis also provided strong evidence for the discriminative power of GM activation patterns, suggesting that altered posterior-chain activation may be one feature observed in this SIS sample. This pattern suggests that altered GM and LD activation may represent a group-level neuromuscular feature of SIS rather than a direct linear marker of symptom intensity. In cross-sectional pain conditions, EMG differences may capture underlying motor-control adaptation or compensatory recruitment patterns that distinguish patients from controls even when they do not vary proportionally with pain, disability, or ROM within the affected group [31,52]. Accordingly, the high discriminative performance observed here should be interpreted as evidence of group separation, not as proof that greater EMG abnormality necessarily reflects more severe clinical presentation. This distinction is important because classification analyses assess group separation, whereas correlations assess within-group linear association; therefore, strong discrimination can coexist with weak correlations.

In the present study, the extremely limited shoulder joint mobility found in participants in the SIS group is consistent with widely reported restrictions in flexion, abduction, and rotation secondary to pain avoidance, capsular tightening, impaired scapular activity, and rotator cuff inhibition [26,53,54]. These mobility losses, together with altered neuromuscular control, can establish a vicious cycle that strengthens dysfunctional movement patterns. The absence of strong correlations between ROM deficits and EMG measurements implies that mobility deficits can operate independently of POS motor deficits through a more complex nonlinear interaction.

Clinically, these findings potentially support the integration of kinetic chain–informed rehabilitation strategies for patients with SIS. Although traditional shoulder-focused rehabilitation remains foundational, evidence increasingly supports incorporating lumbopelvic stability, hip extensor strengthening, and trunk control training to optimize shoulder function. The present findings suggest that lumbopelvic factors may be worth considering in future rehabilitation research for SIS. Future intervention studies are needed to determine whether rehabilitation strategies that include GM or LD training offer any advantage over standard shoulder-focused rehabilitation.

The limitations of this study include its cross-sectional design, which does not allow for causal inference of the results. Second, participants were limited to sedentary young adults with a BMI below 30 kg/m^2^. Although this improved sample homogeneity, it also substantially limits the external validity of the findings, and the results should therefore be interpreted as applicable only to a similar population. Third, surface EMG cannot completely isolate the deep muscle effects or reduce crosstalk, despite standard electrode positioning and filter settings. Future studies should investigate the longitudinal effects of kinetic chain-based rehabilitation and determine whether correcting POS dysfunction leads to clinically significant improvements in shoulder mechanics, pain, and function. Research integrating 3D motion analysis, scapular kinematic assessment, and myofascial imaging could clarify how lumbopelvic deficits contribute to shoulder pathology. Another limitation is the lack of objective control for physical activity levels, which may have introduced unmeasured variability in the outcomes. Additionally, although the asymmetry in the side assessed by LD does not constitute bias, it could modestly limit the generalizability of our findings and is noted here for transparency. Finally, the sample size of 60 participants was adequate for detecting the planned between-group differences, but it remains relatively modest for multivariable regression analyses involving several variables. Therefore, the predictive models should be interpreted cautiously, and future studies with larger samples are needed to confirm the stability and generalizability of these findings.

## 5. Conclusions

In this study of young adults with subacromial pain, differences in gluteus maximus and latissimus dorsi sEMG activity were observed compared with healthy controls. These findings suggest an association between subacromial pain and altered activation patterns in muscles related to the posterior oblique sling. However, the present cross-sectional design does not allow causal inference, and the diagnostic or prognostic value of these measurements remains to be established in larger prospective and validation studies.

## Data Availability

The data are available from the corresponding author upon reasonable request.

## Abbreviations

The following abbreviations are used in this manuscript:

SIS	Subacromial impingement syndrome
VAS	Visual analog scale
NPRS	Numeric Pain Rating Scale
NPS	Numeric pain scale
QuickDASH	Quick Disabilities of the Arm, Shoulder, and Hand questionnaire
ROM	Range of motion
EMG	Electromyography
sEMG	Surface electromyography
RMS	Root mean square
MVIC	Maximum voluntary isometric contraction
BMI	Body mass index
EHI	Edinburgh Handedness Inventory
POS	Posterior oblique sling
GM	Gluteus maximus
LD	Latissimus dorsi
SD	Standard deviation
MD	Mean difference
CI	Confidence interval
OR	Odds ratio
ROC	Receiver operating characteristics
AUC	Area under the curve
VIF	Variance inflation factor

## References

[1] Lucas J., Van Doorn P., Hegedus E., Lewis J., Van Der Windt D. (2022). A Systematic Review of the Global Prevalence and Incidence of Shoulder Pain. BMC Musculoskelet. Disord..

[2] Hoppe M.W., Brochhagen J., Tischer T., Beitzel K., Seil R., Grim C. (2022). Risk Factors and Prevention Strategies for Shoulder Injuries in Overhead Sports: An Updated Systematic Review. J. Exp. Orthop..

[3] Creech J.A., Silver S. (2025). Shoulder Impingement Syndrome. StatPearls.

[4] Van Der Molen H.F., Foresti C., Daams J.G., Frings-Dresen M.H.W., Kuijer P.P.F.M. (2017). Work-Related Risk Factors for Specific Shoulder Disorders: A Systematic Review and Meta-Analysis. Occup. Environ. Med..

[5] Ge M., Zhang Y., Li Y., Feng C., Tian J., Huang Y., Zhao T. (2022). Publication Trends and Hot Spots in Subacromial Impingement Syndrome Research: A Bibliometric Analysis of the Web of Science Core Collection. J. Pain Res..

[6] Jafarian Tangrood Z., Spontelli Gisselman A., Sole G., Cury Ribeiro D. (2023). Clinical Course of Pain and Function in Subacromial Shoulder Pain: A Systematic Review with Meta-Analysis. Phys. Ther. Rev..

[7] Witten A., Clausen M.B., Thorborg K., Hölmich P., Barfod K.W. (2025). The Challenge of Diagnosing Patients Presenting with Signs and Symptoms of Subacromial Pain Syndrome: A Descriptive Study of 741 Patients Seen in a Secondary Care Setting. Orthop. J. Sports Med..

[8] Garving C., Jakob S., Bauer I., Nadjar R., Brunner U.H. (2017). Impingement Syndrome of the Shoulder. Dtsch. Ärztebl. Int..

[9] Amin A., Kashif M., Jan S.I.U., Shoukat T., Zulfiqar K., Taimoor M. (2025). Association of Intrascapular Myofascial Trigger Points and Thoracic Hypokyphosis with the Risk of Development of Shoulder Impingement Syndrome: A Cross-Sectional Study. Heal. J. Physiother. Rehabil. Sci..

[10] Telli H., Sağlam G. (2023). Scapular Dyskinesis and Loss of Cervical Lordosis in Myofascial Pain Syndrome and Its Effects on Pain and Posture Disorders. Turk. J. Phys. Med. Rehabil..

[11] Villa Muñoz T., Velázquez Saornil J., Sánchez Milá Z., Romero-Morales C., Almazán Polo J., Baraja Vegas L., Hugo Villafañe J., Abuín-Porras V. (2024). Comparative Evaluation of the Efficacy of Therapeutic Exercise versus Myofascial Trigger Point Therapy in the Treatment of Shoulder Tendinopathies: A Randomised Controlled Trial. BMJ Open Sport. Exerc. Med..

[12] Joseph L.H., Pirunsan U., Sitilertpisan P., Paungmali A. (2017). Effect of Lumbopelvic Myofascial Force Transmission on Glenohumeral Kinematics—A Myo-Fascia-Biomechanical Hypothesis. Pol. Ann. Med..

[13] Myers T.W. (2021). Anatomy Trains: Myofascial Meridians for Manual Therapists and Movement Professionals.

[14] Sušic A., Bankin V., Hoster J., Zokalj M. (2020). Approach to Understanding of Biomechanical Locomotion System. Interdiscip. Descr. Complex. Syst..

[15] Vleeming A., Pool-Goudzwaard A.L., Stoeckart R., van Wingerden J.P., Snijders C.J. (1995). The Posterior Layer of the Thoracolumbar Fascia. Its Function in Load Transfer from Spine to Legs. Spine.

[16] Lim W. (2023). The Influence of Passive Extensibility of the Posterior Oblique Sling’s Upperportion on Contralateral Knee Extension. Physiother. Quart..

[17] Kim J., Kang M., Oh J. (2014). Patients With Low Back Pain Demonstrate Increased Activity of the Posterior Oblique Sling Muscle During Prone Hip Extension. PM&R.

[18] McDonnell M.K., Sahrmann S.A., Van Dillen L. (2005). A Specific Exercise Program and Modification of Postural Alignment for Treatment of Cervicogenic Headache: A Case Report. J. Orthop. Sports Phys. Ther..

[19] Lee J.-K., Hwang J.-H., Kim C.-M., Lee J.K., Park J.-W. (2019). Influence of Muscle Activation of Posterior Oblique Sling from Changes in Activation of Gluteus Maximus from Exercise of Prone Hip Extension of Normal Adult Male and Female. J. Phys. Ther. Sci..

[20] Oliver G.D. (2014). Relationship between Gluteal Muscle Activation and Upper Extremity Kinematics and Kinetics in Softball Position Players. Med. Biol. Eng. Comput..

[21] Paksoy A., Akgün D., Gebauer H., Karczewski D., Lacheta L., Tokish J.M., Chamberlain A., Moroder P. (2024). The Latissimus Dorsi Creates a Dynamic Track for the Inferior Angle of the Scapula during Arm Abduction in Humans. J. Orthop. Surg. Res..

[22] Chan K., Langohr G.D.G., Athwal G.S., Johnson J.A. (2022). The Biomechanical Effectiveness of Tendon Transfers to Restore Rotation after Reverse Shoulder Arthroplasty: Latissimus versus Lower Trapezius. Shoulder Elb..

[23] Hodgetts C.J., Leboeuf-Yde C., Beynon A., Walker B.F. (2021). Shoulder Pain Prevalence by Age and within Occupational Groups: A Systematic Review. Arch. Physiother..

[24] Tauqeer S., Arooj A., Shakeel H. (2024). Effects of Manual Therapy in Addition to Stretching and Strengthening Exercises to Improve Scapular Range of Motion, Functional Capacity and Pain in Patients with Shoulder Impingement Syndrome: A Randomized Controlled Trial. BMC Musculoskelet. Disord..

[25] Richardson E., Lewis J.S., Gibson J., Morgan C., Halaki M., Ginn K., Yeowell G. (2020). Role of the Kinetic Chain in Shoulder Rehabilitation: Does Incorporating the Trunk and Lower Limb into Shoulder Exercise Regimes Influence Shoulder Muscle Recruitment Patterns? Systematic Review of Electromyography Studies. BMJ Open Sport. Exerc. Med..

[26] Gunay Ucurum S., Ozer Kaya D., Kayalï Y., AskïN A., Agah TekïNdal M. (2020). Comparison of Different Electrotherapy Methods and Exercise Therapy in Shoulder Impingement Syndrome: A Prospective Randomized Controlled Trial. Acta Orthop. Traumatol. Turc..

[27] Lanza M.B., Balshaw T.G., Massey G.J., Folland J.P. (2018). Does Normalization of Voluntary EMG Amplitude to MMAX Account for the Influence of Electrode Location and Adiposity?. Scand. Med. Sci. Sports.

[28] Amarantini D., Bru B. (2015). Training-Related Changes in the EMG–Moment Relationship during Isometric Contractions: Further Evidence of Improved Control of Muscle Activation in Strength-Trained Men?. J. Electromyogr. Kinesiol..

[29] Kinsella R., Pizzari T. (2017). Electromyographic Activity of the Shoulder Muscles during Rehabilitation Exercises in Subjects with and without Subacromial Pain Syndrome: A Systematic Review. Shoulder Elb..

[30] Houry M., Bonnard M., Tourny C., Gilliaux M. (2023). Kinematic, Electromyographic and Isokinetic Measurements for the Management of Shoulder Subacromial Pain Syndrome: A Systematic Review. Clin. Biomech..

[31] Chester R., Smith T.O., Hooper L., Dixon J. (2010). The Impact of Subacromial Impingement Syndrome on Muscle Activity Patterns of the Shoulder Complex: A Systematic Review of Electromyographic Studies. BMC Musculoskelet. Disord..

[32] Rushton A., Carlesso L.C., Flynn T., Hing W.A., Rubinstein S.M., Vogel S., Kerry R. (2023). International Framework for Examination of the Cervical Region for Potential of Vascular Pathologies of the Neck Prior to Musculoskeletal Intervention: International IFOMPT Cervical Framework. J. Orthop. Sports Phys. Ther..

[33] Oldfield R.C. (1971). The Assessment and Analysis of Handedness: The Edinburgh Inventory. Neuropsychologia.

[34] Alghadir A.H., Anwer S., Iqbal Z.A. (2016). The Psychometric Properties of an Arabic Numeric Pain Rating Scale for Measuring Osteoarthritis Knee Pain. Disabil. Rehabil..

[35] Kennedy C.A., Beaton D.E., Smith P., Van Eerd D., Tang K., Inrig T., Hogg-Johnson S., Linton D., Couban R. (2013). Measurement Properties of the QuickDASH (Disabilities of the Arm, Shoulder and Hand) Outcome Measure and Cross-Cultural Adaptations of the QuickDASH: A Systematic Review. Qual. Life Res..

[36] Hudak P.L., Amadio P.C., Bombardier C., Beaton D., Cole D., Davis A., Hawker G., Katz J.N., Makela M., Marx R.G. (1996). Development of an Upper Extremity Outcome Measure: The DASH (Disabilities of the Arm, Shoulder, and Head). Am. J. Ind. Med..

[37] Alnahdi A.H. (2021). Validity and Reliability of the Arabic Quick Disabilities of the Arm, Shoulder and Hand (QuickDASH-Arabic). Musculoskelet. Sci. Pract..

[38] Rigoni M., Gill S., Babazadeh S., Elsewaisy O., Gillies H., Nguyen N., Pathirana P.N., Page R. (2019). Assessment of Shoulder Range of Motion Using a Wireless Inertial Motion Capture Device—A Validation Study. Sensors.

[39] Shepherd J., Hansjee S., Divall P., Raval P., Singh H. (2024). How Do Digital Range of Motion Measurement Devices ‘Measure-up’ to Traditional Goniometry in Assessing Shoulder Range of Motion? A Systematic Review and Meta-Analysis. Shoulder Elb..

[40] Hermens H.J., Freriks B., Disselhorst-Klug C., Rau G. (2000). Development of Recommendations for SEMG Sensors and Sensor Placement Procedures. J. Electromyogr. Kinesiol..

[41] Jung E., An D., Yoo W., Kim T., Park I., Oh J. (2022). Comparison of Pelvic Rotation Angle and Electromyography Activity of the Trunk and Gluteus Maximus Muscles during Four Pilates Exercises. J. Musculoskelet. Sci. Technol..

[42] Sugaya T., Sakamoto M., Nakazawa R., Wada N. (2016). Relationship between Spinal Range of Motion and Trunk Muscle Activity during Trunk Rotation. J. Phys. Ther. Sci..

[43] Hoshizaki T., Clancy E.A., Gabriel D.A., Green L.A. (2020). The Reliability of Surface EMG Derived Motor Unit Variables. J. Electromyogr. Kinesiol..

[44] Wang L., Tao H., Chen Q., Qiao M., Song X., Niu W. (2024). Effect of Fatigue on Intermuscular EMG-EMG Coupling during Bench Press Exercise at 60% 1RM Workload in Males. Front. Hum. Neurosci..

[45] Marson R.A., De Sousa N.M.F., Scoz R.D., Mendes J.J.B., Ferreira L.M.A., De Melo M.A.A., Baldissera V., Freitas L.F., Amorim C.F. (2023). Different Smoothing Window Lengths Can Estimate the Neuromuscular Fatigue Threshold at The Same Intensity of the Lactate Threshold During the Leg Press Exercise. Open Sports Sci. J..

[46] Shin S., Kim T., Yoo W. (2013). Effects of Various Gait Speeds on the Latissimus Dorsi and Gluteus Maximus Muscles Associated with the Posterior Oblique Sling System. J. Phys. Ther. Sci..

[47] Zieliński G. (2025). Effect Size Guidelines for Individual and Group Differences in Physiotherapy. Arch. Phys. Med. Rehabil..

[48] Lee W. (2024). Correlation between Gluteus Maximus Strength and Pelvic Rotation Angle during Prone Hip Extension. J. Musculoskelet. Sci. Technol..

[49] Wen M., Hu X., Bao G. (2025). Scapular Dyskinesis-Based Exercise Therapy versus Multimodal Physical Therapy for Subacromial Impingement Syndrome in Young Overhead Athletes with Scapular Dyskinesis: A Randomized Controlled Trial. BMC Sports Sci. Med. Rehabil..

[50] Mise T., Kurita T., Kamakari S., Akuzawa H., Oshikawa T., Matsunaga N., Kaneoka K. (2024). Does Scapular Dysfunction Alter Scapular Muscles Activity and Kinematics during Swim Stroke Motion on Adolescent Swimmers?. Sports Biomech..

[51] Kararti C., Özüdoğru A., Basat H.Ç., Özsoy İ. (2025). Favorable Clinical Outcomes After Humeral Head Depressor Muscle Coactivation Training with EMG for Patients with Arthroscopic Rotator Cuff Repair: A Randomized Controlled Trial. Sports Health A Multidiscip. Approach.

[52] Kolk A., Overbeek C.L., De Witte P.B., Canete A.N., Reijnierse M., Nagels J., Nelissen R.G.H.H., De Groot J.H. (2021). Kinematics and Muscle Activation in Subacromial Pain Syndrome Patients and Asymptomatic Controls. Clin. Biomech..

[53] Zhong Z., Zang W., Tang Z., Pan Q., Yang Z., Chen B. (2024). Effect of Scapular Stabilization Exercises on Subacromial Pain (Impingement) Syndrome: A Systematic Review and Meta-Analysis of Randomized Controlled Trials. Front. Neurol..

[54] El Melhat A.M., Mohamed H.T.K., Youssef E.F., Gad A.M., Al Hamaky D.M.A. (2025). Identifying Patients with Shoulder Impingement Syndrome Who Improve with Scapular Training: A Clinical Prediction Study. Physiother. Quart..

